# Limits to Plant Species’ Ranges: A Unified Eco-Evolutionary Model and Molecular Mechanisms

**DOI:** 10.3390/plants15203170

**Published:** 2026-10-16

**Authors:** Yan-Wen Lv, Xiao-Han Liu, Hui Xie, Yi-Lan Bai, Zi-Yi Gao, Xin-Sheng Hu

**Affiliations:** 1 College of Forestry and Landscape Architecture, South China Agricultural University, Guangzhou 510642, China; lyw13671474450@163.com (Y.-W.L.); lxiaohan0525@163.com (X.-H.L.); xiehui1721@163.com (H.X.); baiyilann@163.com (Y.-L.B.); ziyigao76916@163.com (Z.-Y.G.); 2 Guangdong Key Laboratory for Innovative Development and Utilization of Forest Plant Germplasm, Guangzhou 510642, China

**Keywords:** range limits, eco-evolutionary model, landscape genomics, genomic structural variation, mating system

## Abstract

The formation of a species’ range generally involves dispersal and selection processes where dispersal enhancing species expansion is counterbalanced by selection that reduces mean population fitness at the edge of a species’ range. The application of high-throughput sequencing has made it possible to identify the genetic basis for plant adaptation, which allows for assessing molecular mechanisms of genetic regulation at the edge. Here, we first review the theoretical advances in studying a species’ range based on a unified eco-evolutionary model. Genetic loads generated by different evolutionary processes can be uniformly expressed in a general genetic component of regulation (${\bar{r}}_{g}$). We then discuss the molecular basis of adaptation from recent empirical studies, including SNP-based detection of adaptation signatures, the regulation of gene expression, genomic structural variation, transposable elements, gene family expansion/contraction, and genome size. We also discuss the potential of applying estimates of molecular evolutionary rates (*K_a_*/*K_s_*) to infer plant adaptation in the edge populations by comparing the differences between the central and edge populations or between the central and edge communities. Finally, we discuss how plant mating systems regulate genetic loads in edge populations and alter the relative genetic loads at the gametophyte versus sporophyte phase. By linking the eco-evolutionary model with genomic evidence, this review explores several issues to incorporate genetic and ecological regulations and genomic variation into studies on a species’ range. Addressing these issues can extend our understanding of the limits to a plant species’ range.

## 1. Introduction

Understanding why and how a plant species occupies one region but not others has long been a fundamental goal in plant ecology, evolutionary biology, and conservation sciences [1,2,3]. Plant species vary markedly in the size, continuity, and boundary sharpness of their geographic ranges [4]. Almost all species on Earth have limited geographic distributions. This raises two basic questions: How is a species’ range limited under global climate and environmental changes? What mechanisms maintain them? Answers to these questions may help us to understand the pattern of biodiversity and to develop an effective conservation strategy for a particular species. So far, many theories and empirical studies have tackled this theme, and several specific issues have been recorded [5,6,7]. Although the evolutionary forces driving a species’ range have been studied and discussed in the literature, a general framework, especially covering different genetic loads regulating a species’ range, has not been synthesized in detail. Here we bring together different models of evolutionary processes shaping the mean fitness of a population and synthesize them in a general eco-evolutionary model to describe a species’ range.

The eco-evolutionary explanation of a species’ range goes back to Darwin’s pioneering work [8], who emphasized the roles of climate, other physical conditions, and interspecific interactions in determining a species’ range. This was followed by Haldane [9], who emphasized the joint effects of natural selection and gene flow in determining a species’ range. Gene flow from the central populations constrains adaptation in the edge populations, thereby limiting species expansion, which has been explored in detail [10,11,12]. These two viewpoints provide a basis to examine eco-evolutionary processes underlying a species’ range. Subsequent models have been developed to explore diverse models of selection (ecological niche models) and neutral processes [13]. These models explicitly or implicitly involve the adaptation of edge populations to local environments despite different model assumptions. The eco-evolutionary model is extended from the previously single population-based to the metapopulation-based models and is used to interpret and predict species’ geographic ranges [6,7,14]. The limit of the species distribution marks the transition from species presence to absence and reflects constraints on population adaptation [15,16].

The application of high-throughput sequencing has transformed previous studies on population adaptation, mainly based on phenotypic variation, into the integration of both phenotypic and genome-wide sequence variations. This transition provides new opportunities to examine molecular mechanisms underlying a species’ range [17]. A growing body of work has integrated genome-wide SNP variation, transcriptome-based RNA-seq data, structural variants (SVs), transposable elements (TEs), and molecular evolution analyses to identify adaptation signatures and to clarify the molecular basis of population adaptation. However, the linkage between molecular mechanisms and the eco-evolutionary model has not been explored, which hinders our insights into a general picture of a plant species’ range at a more fundamental level. Here we begin by synthesizing the eco-evolutionary model, which is predominantly applied to interpret animal species’ ranges, and unify the mean Malthusian fitness of the population for interpreting plant species’ ranges. We then review recent advances in molecular mechanisms related to a species’ range and connect the empirical findings to population fitness in the eco-evolutionary model, including adaptation signature detection in terms of various genomic markers, molecular evolution and the regulation of plant mating systems on a species’ range. Finally, we discuss a few important issues, and their exploration can expand our understanding of the molecular mechanisms underlying the formation of a plant species’ range.

## 2. A Unified Eco-Evolutionary Model

Without loss of generality, consider a population size *N* as a continuous variable. In one-dimensional and homogeneous space, the general framework for an eco-evolutionary model for population dynamics at the edge of a plant species’ range can be synthesized in the diffusion–reaction process (Fisher-KPP equation):

(1) $$\frac{\partial N}{\partial t}=\bar{r}N+\frac{\sigma^{2}}{2}\frac{\partial^{2}N}{\partial x^{2}}$$

where $\bar{r}$ (the reaction term) is the mean Malthusian fitness of the population, *σ*^2^ (the diffusion term) is the variance of distance between parent and offspring, *t* is the time, and *x* is the position in space. Notice that $\bar{r}$ is the potential function of biotic, genetic and abiotic factors [18]. Equation (1) can be derived by combining the changes due to population fitness (selection) and due to dispersal from foreign populations. The first part is a general expression for a population growth change where $\bar{r}$ can be expressed in different forms under various scenarios. The second part is approximated by a diffusion process. These two parts are separately modeled and then combined to yield Equation (1). Although this framework is predominantly applied to animal species’ ranges, the two essential components ($\bar{r}$ and *σ*^2^) have not been understood in a general way, particularly for plant species.

Fisher [19] first proposed this model where $\bar{r}$ was expressed in the logistic growth rate to describe the spread of advantageous genes in a two-dimensional space. The diffusion term in (1) refers to short-distance movement per unit time. Up to now, many variant models have been developed to describe population spatial spread [20,21,22] but have not been synthesized in a general way for a species’ range. A species’ range is limited in theory when the mean Malthusian fitness $\bar{r}$ is not greater than zero. For instance, if $\bar{r}$ is expressed as $\bar{r}(N)=r_{0}N(1-N/K)(N/A-1)$, where *A*, *K*, and *r*_0_ are the Allee-effect threshold [23], the carrying capacity, and the intrinsic growth rate, respectively; a species’ range may be formed when $\bar{r}(N)$ is negative and the diffusion process is weaker. If $\bar{r}(N)$ is the per capita growth rate (without Allee effects), the edge population grows, and the species expands across space as a traveling wave under the conditions of $\bar{r}(N)>0$, $\bar{r}(K)=0$, and $\partial \bar{r}(N)/\partial N<0$. At a low density where $\bar{r}(0)$ approaches *r*_0_, the front wave moves at an approximate speed of $c=\sqrt{2\bar{r}(0)\sigma^{2}}$ [19,24].

**Figure 1:**
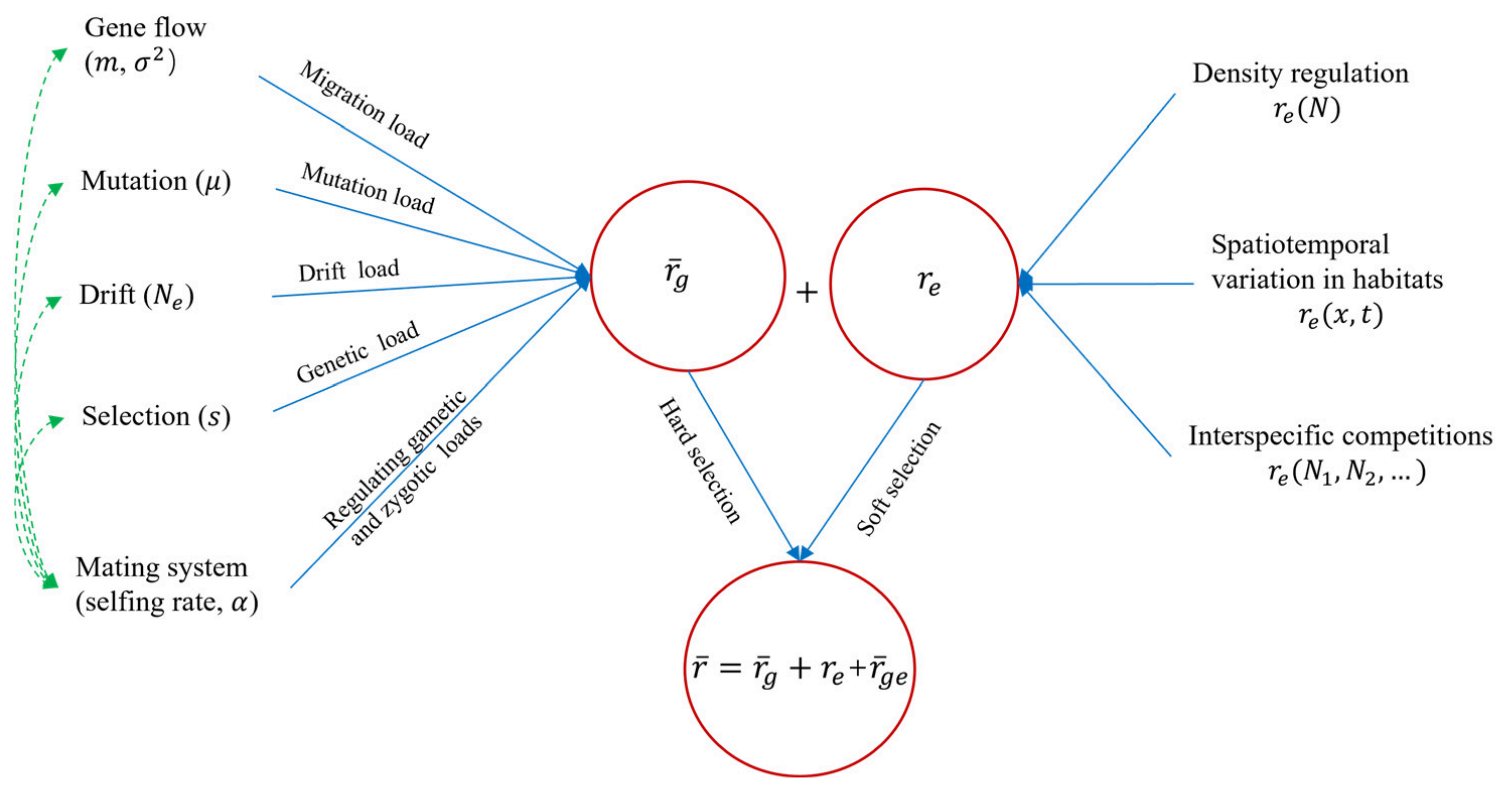
The potential eco-evolutionary processes affecting the mean Malthusian fitness of a population ($\bar{r}$). The $\bar{r}$ is decomposed into the components of *r_e_*, ${\bar{r}}_{g}$, and the genotype-by-environment ${\bar{r}}_{\mathrm{ge}}$. Here *r_e_* is considered to be mainly related to soft selection, where the number of survivors depends on individual competition ability (among intra- or interspecific individuals) and the genotypes with relatively higher advantages have larger survival probabilities. The ${\bar{r}}_{g}$ is considered to be mainly related to hard selection, where the number of survivors depends on their adaptation to the environment rather than the density of competitors. The genetic component (${\bar{r}}_{g}$) may come from genetic loads from gene flow, mutation, drift and selection. The mating system affects the mean Malthusian fitness via interactions with basic evolutionary forces. The unidirectional arrows in the figure indicate causal relationships, while the two-way arrows indicate mutual interaction relationships.

In the following discussions of theoretical models, we assume that Allee effects on $\bar{r}$ are negligible except for the discussions on potential Allee effects on genetic drift. Next, we synthesize the $\bar{r}$ function under different eco-evolutionary processes, with an emphasis on different genetic loads (Figure 1). These processes can generate a mean Malthusian fitness deviating from the uniform per capita logarithmic growth rate under Malthusian law [18]. The mean Malthusian fitness ($\bar{r}$) can be decomposed into the genetic (${\bar{r}}_{g}$) and ecological (*r_e_*) components and their potential genotype-by-environment interactions (${\bar{r}}_{\mathrm{ge}}$). They may unequally limit species’ ranges, although their relative effects could vary with species.

Equation (1) indicates that a species’ range is shaped by the change in the mean Malthusian fitness of the edge population ($\bar{r}$). Selection interacts with other eco-evolutionary processes within species (e.g., among genotypes or genes) or between species to alter $\bar{r}$ at the edge. With Haldane’s viewpoint, migration load weakens local adaptation [25,26] and facilitates species limitation by reducing $\bar{r}$ at the edge. Suppose that the Malthusian population fitness is regulated by the logistic growth model (intraspecific competition) and is associated with an adaptive quantitative trait. The mean Malthusian fitness $\bar{r}$ is expressed in terms of an adaptive phenotypic trait *z* that changes along an environmental gradient, the components of the ecological term *r_e_*(*N*) (soft selection) and the genetic term ${\bar{r}}_{g}(z)$ (hard selection) in $\bar{r}$ are written as

(2) $$\bar{r}=\mathrm{rN}\left(1-\frac{N}{K}\right)-\frac{V_{P}}{2V_{s}}-\frac{{\left(\bar{z}-z_{\mathrm{opt}}\right)}^{2}}{2V_{s}}$$

where *V_P_* is the phenotypic variance of the trait, $\bar{z}$ is the population mean of the trait, *z*_opt_ is the trait optimum, and *V_s_* is the inverse of selection strength [27]. This decomposition does not include a potential regulation from eco-genetic interaction, denoted by ${\bar{r}}_{\mathrm{ge}}$ (phenotypic plasticity). This interaction likely contributes to regulating a species’ range. Also, the relative contributions of ecological vs. genetic regulations (*rₑ*(*N*) vs. ${\bar{r}}_{g}(z)$) to limit a species’ range remain to be clarified. At equilibrium, the ecological and genetic regulation counterbalances each other, yielding $\bar{r}=0$, and the range boundary is restricted along the gradient of the trait. When the environmental gradient is steep relative to dispersal distance, the equilibrium range boundary is distributed closer to central populations, resulting in a narrower spatial distribution [10,27,28]. Several theories address how the joint effects of natural selection and gene flow affect the stability of species ranges [10,29,30].

Suppose that the mean Malthusian population fitness $\bar{r}$ is controlled by the ecological regulation *r_e_*(*N*) and genetic drift effects (*N_e_*). Genetic drift shapes a species’ range via interaction with selection to regulate $\bar{r}$. Theoretical studies show that range expansion depends on whether genetic drift erodes genetic variation below the level needed for adaptation to new environmental conditions [31,32]. Genetic drift at the edge generates drift load by shifting population fitness away from its optimum. This process reduces $\bar{r}$ [31,33], which is represented by independent white noise *dw*:

(3) $$\bar{r}=r_{e}(N)+N^{-1/2}\mathrm{dw}$$

where *N*^−1/2^ is the strength of the drift effect. Notice that the white noise *dw* is not correlated in time and space, with a mean of zero and an expectation of autocorrelation being the Dirac delta function. A range size can be estimated from population density distribution. Genetic drift constrains range expansion indirectly by reducing the efficacy of selection against deleterious mutations, thereby increasing mutation load at the edge and limiting a species’ range [34,35]. Empirical evidence supports this expectation in *Arabidopsis lyrata* subsp. *lyrata* [36]. The *r_e_*(*N*) reinforces impacts on small edge populations if Allee effects are present. If ecological regulation weakens Allee-effect constraints, it can alleviate the processes that limit range expansion. Du et al. [37] showed that individuals at the edge could maintain certain population density via shifting the boundary forward or backward, thereby avoiding Allee-effect limitation and promoting expansion. By contrast, when genetic drift overwhelms natural selection, species ranges may contract from the edge or undergo abrupt fragmentation, even when environmental change is relatively gradual [38].

Suppose that the mean Malthusian population fitness $\bar{r}$ is regulated by ecological regulation *r_e_*(*N*) and deleterious mutant genes. Mutation can interact with selection to regulate $\bar{r}$ via mutation load. According to Haldane [39] and Kimura [40], the mutation load at a single locus with mutation rate *μ* is equal to *μ*. When there are *n* loci with deleterious mutants across whole genomes, the overall mutation load is equal to *L* = *nμ*. The genetic component of regulation in the Malthusian fitness ${\bar{r}}_{g}$ is expressed by ${\bar{r}}_{g}=\ln\left(1-L\right)\approx -L$. Thus, $\bar{r}$ is given by

(4) $$\bar{r}=r_{e}\left(N\right)-\mathrm{n\mu}$$

Once the mutation load exceeds the ecological regulation at the edge, $\bar{r}$ is negative and population expansion at the edge is blocked. In addition, when genetic drift interacts with mutation, the selection efficacy decreases and mutation load increases in edge populations, as reported in birds [41]. Simulations also show that mutation load can change dynamically when environmental change shifts species ranges [42]. Empirical studies support this prediction, such as in *Arabidopsis lyrata* [36,43] and *Vitis arizonica* [44].

Suppose that the mean Malthusian population fitness $\bar{r}$ is only regulated by the ecological regulation arising from temporal fluctuation. Here, $\bar{r}$ is the time-continuous Malthusian rate. Then, $\bar{r}$ is expressed as $\bar{r}=r_{e}(N)$. In homogeneous space, the edge populations with $\bar{r}>0$ tend to spread in space in a traveling wave [20]. However, temporal variation in habitat reduces genetic diversity and weakens the maintenance of evolutionary potential due to competition among individuals, thereby limiting population adaptation to diverse habitats. Habitats in the edge populations often tend to fluctuate more temporally than the habitats in the central populations [45,46,47], and such temporal dynamics affect both ecological and evolutionary processes, ultimately restricting spatial distributions. Holt et al. [48] showed that temporal variation in habitats can shift the long-term growth rate in a directional way. If *r*(*t*, *x*) denotes the Malthusian population growth rate in environment *x* at time *t*, then the mean Malthusian growth rate under environmental variation over a time period *T* is calculated as:

(5) $${\bar{r}}_{e}\left(x\right)=\frac{1}{T}\int_{0}^{T}r(t,x)\;\mathrm{dt}$$

Temporal dynamics can drive either range contraction or range expansion. Benning et al. [49] examined population spread along spatial environmental gradients and found that temporal environmental variation has a critical threshold, above which range expansion is prevented and a stable range forms.

The mating system as a life-history trait interacts with basic evolutionary forces to shape population dynamics and hence affect a species’ range. The mating system directly alters the diffusion coefficient *σ*^2^ for diploid dispersal via seeds only ($\sigma^{2}=\sigma_{\mathrm{seed}}^{2}$) and gene flow via both seed and pollen flow ($\sigma^{2}=\sigma_{\mathrm{seed}}^{2}+\frac{1-\alpha }{2}\sigma_{\mathrm{pollen}}^{2}$) [50], which differs from most animal species. Selfing discounts foreign pollen and thereby alters the spatial spread of both adaptive and neutral genes [51]. If the mating system has only weak effects on intra-competition, *r_e_*(*N*) can be approximated by the case under outcrossing, but ${\bar{r}}_{g}$ remains to be explored. Suppose that the mean Malthusian population fitness $\bar{r}$ is regulated by both the ecological regulation and genetic loads in a mixed mating system. When the population growth is affected by an adaptive phenotypic trait (*z*), selfing directly interacts with gametic selection but indirectly with zygotic selection, thereby changing mean population fitness. Hu et al. [52] derived ${\bar{r}}_{g}$ as follows:

(6) $${\bar{r}}_{g}=-\alpha \beta \left(\frac{1}{2}V_{\max}+\frac{1}{4}z_{\mathrm{opt}}^{2}\right)S-\left(1-\alpha \right)\beta \left(\frac{1}{2}V_{A}+\frac{1}{4}{\left(\bar{z}-z_{\mathrm{opt}}\right)}^{2}\right)S-\left(\frac{2}{2-\alpha }V_{A}+{\left(\bar{z}-z_{\mathrm{opt}}\right)}^{2}\right)S$$

where *β* is the ratio of selection coefficients between gametic and zygotic stages, *S* is the selection coefficient at the zygotic stage, *V_A_* is the additive genetic variance, and *V*_max_ is the maximum additive variance. Selfing restricts a species’ range under gametic selection only but promotes range expansion under zygotic selection only. This theory implies a condition for the common view that species with self-fertilization often have wider ranges than outcrossing species [52].

Note that some species possess a partial selfing system in the edge populations that differ from the predominantly outcrossing system in the central populations [13]. Selfing interacts with genetic drift and mutation to reduce the effective population size (*N_e_*) and weaken the efficacy of gametic selection, thereby favoring the retention of deleterious alleles and increasing genetic load [53]. This process can further amplify ${\bar{r}}_{g}$, and impede population expansion, particularly when the edge populations are small.

Darwin [8] argued that a species’ geographic distribution is shaped by climate and environmental conditions as well as by interspecific competition. Suppose that the mean Malthusian population fitness $\bar{r}$ is only regulated by the ecological regulation arising from interspecific competition. When interspecific competition is incorporated, *r_e_* is constructed by following the classical Lotka [54]–Volterra [55] model:

(7) $$r_{e}\left(N_{1},N_{2}\right)=r_{1}\left(1-\frac{N_{1}}{K_{1}}-\frac{aN_{2}}{K_{1}}\right)$$

Here, *r_e_*(*N*_1_, *N*_2_) is the ecological component of the mean Malthusian growth rate of species 1 under the influence of species 2; *N*_1_ and *N*_2_ are the population sizes of species 1 and species 2, respectively; *a* is the competitive effect of species 2 on species 1; and *K*_1_ is the carrying capacity of species 1. When $\bar{r}$ is affected by an adaptive trait *z*, the competition coefficient *a* can be expressed as a function of interspecific differences in *z*, whereas ${\bar{r}}_{g}(z)$ follows the genetic term in (2). Equation (7) can be extended to the case of multispecies competition. Case & Taper [56] developed a theory of species distribution under two-species interactions, showing that biotic interactions can shape species’ ranges. Empirical evidence is recorded on the impacts of interspecific competition on species’ ranges [57,58,59,60]. Some theoretical models further include biotic gradients and environmental factors in the ecological regulation term, such as *r_e_*(*N*_1_, *N*_2_, *f*), where *f* denotes an environmental factor. Alexander et al. [60] assumed an environment that was composed of one biotic and one abiotic variable, with the species’ fitness determined by a trade-off between responses to biotic and abiotic stresses. Their model showed that the genotypes favored at the edge may depend on the biotic environmental context and the form of the fitness trade-off.

Note that the general framework (1) needs revisions when plant populations spread along heterogeneous environments. The diffusion coefficient *σ*^2^ could be adjusted to *σ*^2^(*x*), allowing dispersal to vary across environmental locations. The ecological and genetic regulatory components are written as *r_e_*(*N*, *x*) and ${\bar{r}}_{g}(x)$, respectively, since both change with population position (*x*). Similarly, the long-distance dispersal moves the model beyond the diffusion approximation but still can be treated as an extension of the diffusion–reaction framework [20].

When the metapopulation dynamics are considered in the diffusion–reaction framework, the local population sizes are no longer fixed but fluctuate. Szep et al. [61] used Wright’s island model and assumed multilocus linkage equilibrium to derive the joint density *ψ*[*N*, *p*] of population size (*N*) and multilocus allele frequency (*p*), under the joint effects of ecological regulation *r*(*N*) and genetic regulation *r_g_*(*p*). Barton [14] subsequently expressed the genetic component as a function of the mean adaptive phenotypic trait *r_g_*($\bar{z}$) and obtained the joint density *ψ*[*N*, $\bar{z}$] for evaluating species ranges.

Although the preceding framework is well established and can be unified in a general diffusion–reaction form, its connection to empirical molecular studies is scarce. The next section reviews the advances in empirical studies in this area and discusses how these studies connect to the unified eco-evolutionary model.

## 3. Molecular Bases for Adaptation in Plant Spatial Distribution

### 3.1. SNP-Based Detection of Adaptation Signatures

A species’ range could be maintained jointly by selection and other neutral processes (gene flow, genetic drift, and mutation). When *r_e_* is low, ${\bar{r}}_{g}$ determined by selective loci is then dominant. Identification of selective loci provides the genetic basis for insights into genetic regulation. Based on the diffusion–reaction framework, gene flow may swamp locally adaptive alleles when populations are investigated across a spatial scale smaller than the characteristic cline length, $l=\sqrt{\sigma^{2}/2s}$, where *s* is the selection coefficient of adaptive alleles [62], unless selection strength is very strong at some loci (*s*≫*σ*^2^/2) or the cline is only recently formed. Across spatial scales larger than *l*, local populations are more likely to possess alleles adapted to different habitats. Neutral processes facilitate the generation of linkage disequilibrium (LD) among loci, which reduces the power of detecting selective loci. Genome-wide scans for adaptive signatures can be performed with population genomic analysis, such as *F_st_* outliers where local adaptation potentially occurs [63]. However, this approach often generates false-positive loci associated with neutral processes [64]. Furthermore, the environmental variables driving the observed patterns cannot be accurately identified [65]. Therefore, a landscape genomic analysis that combines population genomic analysis with environmental associations is now preferred to identify selective loci [66].

Table 1 summarizes recent studies with multiple approaches to identify adaptive loci and their correlated environmental gradients in different tree and crop species. The genomic datasets involved were SNPs derived from restriction site-associated DNA sequencing (RAD), genotyping-by-sequencing (GBS), whole-genome sequencing (WGS), transcriptome sequencing (RNA-seq), and single enrichment primer technology (SPET). When the SNPs associated with geographic or environmental factors are identified, the adaptation signatures could occur at corresponding loci. The basic procedure for such analyses follows detection of SNPs, annotation of candidate genes linked with the SNP variants and identification of functional pathways involving local adaptation. For example, there were 136 SNPs identified in Tibetan Plateau populations of *Hordeum vulgare* and 447 annotated candidate genes, including HvSs1 and HvVrn-H1, which were associated with cold-stress responses and flowering-time regulation [67]. Several candidate genes related to climate adaptation were identified in *Picea rubens*. Functional annotation and GO enrichment analyses indicated that these genes were mainly involved in drought stress, heat stress, cold stress, phenology regulation, and DNA/RNA repair pathways, including NBR1, HSFB-2B, and FPA [68].

Correlations between gene frequency and environmental gradients strongly imply local adaptation [69,70]. Ample evidence supports this implication, such as a latitudinal shift in PtFT2 allele frequency in *Populus tremula* [71], or associations of allele frequencies with temperature and precipitation in *Fagus sylvatica* [72]. However, when phenotypic traits change continuously along environmental gradients, the relationship between the environmental factors and the associated allele frequencies may not always show a simple monotonic cline, partly because of phenotypic plasticity (*r_ge_*). Lotterhos [73] showed that different mutations reached similar multivariate phenotypic optima in different regions of a landscape. This could imply that similar adaptive outcomes could be achieved through different genetic pathways across geographic regions. Similar evidence to this was also reported in *Pinus contorta* [74].

Because gene flow blurs local genetic differences, the range covered by the investigated populations may affect the power of detecting adaptive loci [75]. In *Quercus lobata*, Gugger et al. [76] observed that adaptive loci were detected at multiple spatial scales, implying that independent selective processes could potentially act on these loci under different genetic or environmental backgrounds. In contrast, there were large differences in detecting selective loci between spatial scales in *Eucalyptus regnans* [75]. This was likely related to microgeographic habitat heterogeneity where local selection strength exceeded the homogenizing effect of gene flow [69,77,78]. Nevertheless, the strength of microenvironmental adaptation could depend on ecological and genetic contexts and may vary among species and landscape types [79].

Reports in the literature indicate that species with different degrees of genetic relatedness had common genes or nearby genomic regions displaying local adaptation across their natural distribution ranges. Such a phenomenon may reflect convergent adaptive responses to similar environmental pressures. More broadly, the same genetic loci are repeatedly involved in local adaptation across populations or species [80,81,82], such as in *Quercus lobata* and its close relative *Q. rugosa* [76] and in *Eucalyptus* species [83]. These adaptive loci did not correlate with species range sizes and essentially did not support the hypothesis that geographic range size is associated with the species’ responses to climate change [84]. Whiting et al. [82] also observed common patterns of adaptation among twenty-five distantly related plant species. Further work is needed to clarify whether locally adaptive loci and related genomic regions generally occur in local adaptation across multiple plant species.

Landscape genomic analysis is applied to separate adaptive loci from the neutral genomic background and to reveal local adaptation signatures at the molecular level. However, it should be noted that high correlations among environmental factors potentially exist, which makes it difficult to disentangle individual effects and limits direct identification of specific selective agents that drive adaptive differentiation among populations [85]. In addition, some studies only considered climatic variables due to limited data availability, and other environmental factors affecting local adaptation were overlooked. Most current studies employ RDA, where parts of the variation remain unexplained [86,87,88]. Under the conditions of strong population structure, conditional neutrality, genetic redundancy, and other complex mechanisms, landscape genomic analysis could fail to detect candidate loci for climate adaptation [81,89].

**Table 1:** Landscape genomic analyses and major drivers of genomic variation in plants.

Species	Data	Method	Variance Component (%)			**Key Environmental Variable**	**Reference**
			Env	Pop	Geo		
*Fagus sylvatica*	RAD-seq	RDA	17.0	-	9.0	Altitude; humidity	[90]
*Quercus longinux*	RAD-seq	BayeScan; PCAdapt; LFMM; pRDA; GF	2.6	-	1.9	Wind; soil	[91]
*Vigna unguiculata*	RAD-seq	LFMM; pRDA; GF	5.0	2.0	3.0	Bio2; Bio3; Bio5	[92]
*Ammopiptanthus mongolicus*	RAD-seq	hierfstat; LFMM; RDA; pRDA; GF	24.0	12.0	4.0	Bio4; Bio5; Bio6; Bio17	[93]
*Pterocarya macroptera*	RAD-seq	PCAdapt; OutFLANK; BayEnv; LFMM; RDA	8.6	-	3.7	Temperature-related variables	[94]
*Hordeum vulgare*	GBS	BayPass; LFMM; RDA; pRDA; sRDA	3.9	15.48	-	Water availability	[95]
*Aegilops tauschii*	GBS	hierfstat; GEA: RDA	57.8	-	5.6	Bio7	[96]
*Eragrostis tef*	WGS	GEA: RDA	0.63	-	-	Bio7; Bio13	[97]
*Castanea dentata*	WGS	*F_st_*; LFMM; RDA; pRDA; GF	1.2	0.8	0.3	TD; DD_18; AHM; Eref; EXT	[98]
*Fragaria nilgerrensis*	WGS	BayPass; RDA	12.25	-	11.58	Bio4; Bio15	[99]
*Acer tsinglingense*	WGS	vegan; LFMM; RDA; GF	38.78	-	63.78	Bio2; Bio7; Bio11	[100]
*Actinidia eriantha*	WGS	vegan; LFMM; pRDA	19.69	0.42	0.14	Prec_03; Srad_03	[101]
*Cephalotaxus oliveri*	RNA-seq	BayeScEnv; RDA	18.6	-	0.4	Bio8; Bio12; soil; EVI; Mg	[86]
*Pseudotaxus chienii*	RNA-seq	BayeScan; LFMM; RDA	0.78	-	11.3	Bio11; Bio14; soil; altitude	[87]
*Picea crassifolia*	RNA-seq	*F_st_*; RDA; pRDA	2.33	-	3.42	Bio4; Bio13; altitude	[102]
*Picea brachytyla*	RNA-seq	*F_st_*; RDA; pRDA	1.27	-	1.32	Bio1; Bio2; Bio12; Bio15	[103]
*Portulaca hatschbachii*	AFLP	BayeScan; RDA; pRDA	26.7	-	-	Temperature; precipitation	[88]
*Taxus baccata*	SPET	BayPass; LFMM; PDA; pRDA; GF	18.1	10.5	13.0	Bio1; Bio2; Bio4; Bio9; Bio12; Bio15	[104]
*Tetrastigma hemsleyanum*	SLAF	BayeScan; SELESTIM; BayEnv; X^T^X; BayPass; RDA; pRDA	27.5	-	2.5	Bio19; PETWeQ	[105]

Note: AFLP: amplified fragment length polymorphism; GBS: genotyping-by-sequencing; GEA: genotype–environment association; RAD-seq: restriction site-associated DNA sequencing; SPET: single-primer enrichment technology; SLAF: specific-locus-amplified fragment sequencing; WGS: whole-genome sequencing; RNA-seq: RNA sequencing; RDA: redundancy analysis; pRDA: partial redundancy analysis; LFMM: latent factor mixed model; GF: gradient forest; Env: environmental effects; Pop: population structure; Geo: geographic effects. Env, Pop, and Geo represent their variance components extracted from the pRDA variance-partitioning analyses reported in the original studies. Symbol “-” indicates that the corresponding component was not reported or was not separately estimated in the original study. Bio1: mean annual temperature (°C); Bio2: mean diurnal range (°C); Bio3: isothermality; Bio4: temperature seasonality (standard deviation × 100); Bio5: maximum temperature of warmest month (°C); Bio6: minimum temperature of coldest month (°C); Bio7: temperature annual range (°C); Bio8: mean temperature of wettest quarter (°C); Bio9: mean temperature driest quarter (°C); Bio11: mean temperature of coldest quarter (°C); Bio12: mean annual precipitation (mm); Bio13: precipitation of wettest month (mm); Bio14: precipitation of driest month (mm); Bio15: precipitation seasonality (coefficient of variation); Bio17: precipitation of driest quarter (mm); Bio19: precipitation of coldest quarter (mm); TD: temperature difference between mean warmest month and mean coldest month (°C); DD_18: degree-days below 18 (°C); AHM: annual heat-moisture index; EXT: extreme maximum temp (°C); Eref: hargreaves reference evaporation (mm); PETWeQ: mean monthly PET of wettest quarter; Prec_03: precipitation of March; Srad_03: solar radiation of March; EVI: enhanced vegetation index.

### 3.2. Regulation by Gene Expression

The gene expression level is a quantitative trait linking phenotype with genotype and is associated with specific genes and genotypes. Variation in gene expression level reflects the effects of the regulatory factors controlling that gene expression. When GWAS is performed with expression data, cis- and trans-regulatory mechanisms can be identified. This analysis provides information alternative to that derived from SNP-based scans of adaptive signatures. Wang et al. [106] provided a comprehensive review on plant gene expression regulation and evolution. The spatiotemporal regulation of gene expression results from long-term evolution and has robust genetic features and regulatory mechanisms (an Evo-Devo feature). However, the gene expression level is also affected by environmental factors and their potential interactions with genotypes (expression plasticity). These features make gene expression analysis at the population level broadly useful in ecology and evolutionary biology. Many studies have employed transcriptome data to examine the effects of environmental changes on gene expression, such as responses to precipitation differences in *Brassica rapa* [107], to seasonal flooding changes in *Ischnosiphon colombianus* [108] and to strong seasonal fluctuations [109].

Suppose that the expression level of a single gene is affected by environmental factors. Consider a common-garden experiment, with *m* sites, *b* genotypes and multiple individual measures for each genotype. Let *z_ijk_* be the expression level of a gene in the *k*-th (*k* = 1, …, *n_ij_*) individual (clone) of the *j*-th (*j* = 1, …, *b*) genotype at the *i*-th (*i* = 1, …, *m*) site or environment. All samples are assumed to be taken from the same tissue at the same developmental stage so that the expression level is comparable across samples biologically. The *z_ijk_* can be partitioned into the mean value (*u*), the site or environment effect (*s_i_*), the genetic effect (*g_j_*), the genotype-by-site effect (*sg_ij_*), and the error term (*e_ijk_*):

(8) $$z_{\mathrm{ijk}}=u+{s_{i}+g}_{j}+{\mathrm{sg}}_{\mathrm{ij}}+e_{\mathrm{ijk}}$$

For a gene with *b* genotypes, (8) is analogous to a multiple-site unbalanced trial where the numbers of observations among sites for each genotype may be unequal. If all factor effects are fixed (fixed model), these effects can be statistically estimated and tested using ANOVA. If partial effects are fixed (e.g., site effects) and other effects are random, these effects can still be estimated and tested using the mixed linear model. In this situation, the genotype-by-site effects are random. Analysis of this model is not further presented here.

Consider a reciprocal-transplant experiment. Suppose that plants with *b*_1_ genotypes at site 1 are reciprocally transplanted with plants with *b*_2_ genotypes. The design is equivalent to a two-site experiment, with *b* (=*b*_1_ + *b*_2_) genotypes at each site. Equation (8) can still be applied to analyze this case. More complicated models can be found in [110,111,112].

Similarly, consider a natural field-sample experiment, with *m* sites, *b* genotypes and unequal numbers of individuals for each genotype. Equation (8) can be treated as either the fixed or the random model to estimate and test different effects, depending on the objectives. The major concern is comparable transcriptome sampling, such as the rigorous control of sampling time (e.g., the same developmental phase) and space (the same position) in field sampling.

Note that most transcriptomic studies measure gene expression from a specific tissue or organ of diploid organisms; therefore, unless single-cell RNA-seq is specified, the gene expression level (e.g., FPKM) represents the average level across cells within the tissue or organ. These datasets rarely separate genotype-specific expression. Therefore, if all samples at a site have the same genotype (*b* = 1), the mean population expression level is a mixture of the habitat or environmental effect, the genetic effect, and the genotype-by-environment effect. Differences in mean population expression level between sites reflect joint variation in these effects [86]. Such mixed-effect differences could approximately represent population-level differences in gene responses to heterogeneous sites. When samples contain different genotypes, the mean population gene expression level is calculated by $\sum_{j=1}^{b}P_{\mathrm{ij}}{\bar{z}}_{\mathrm{ij}.}$, where *P_ij_* is the frequency of the *j*-th genotype in the *i*-th population, and ${\bar{z}}_{\mathrm{ij}.}$ = $\frac{1}{n_{\mathrm{ij}}}\sum_{k=1}^{n_{\mathrm{ij}}}z_{\mathrm{ijk}}$ is the mean expression level. This value deviates from the case described above (*b* = 1). However, this calculation requires separation of *b* genotypes or associated SNPs screened from VCF analysis. A statistical issue for this analysis awaits further completion.

Gene expression is sensitive to environmental changes but genetically controlled and often exhibits expression plasticity (*s* × *g*). Many empirical studies have shown this feature [113,114], such as gene expression plasticity in *Populus lasiocarpa* [115]. An emerging question is to what extent gene expression plasticity arises from adaptive or non-adaptive processes [116,117]. Some experiments indicated that gene expression plasticity was associated with adaptive selection, such as in *Tribolium castaneum* [118]. Gene expression plasticity in rice exhibited different degrees of adaptation under wetland and dryland conditions [119,120].

Table 2 lists a few recent studies where population gene expression varied along environmental gradients, indicating that local adaptation was associated with species’ spatial distribution. The gene expression level was associated with environmental gradients, such as the elevational gradient in *Rhododendron sanguineum* var. *haemaleum* [121], *Boechera stricta* [122] and *Quercus robur* [123].

In theory, the central and edge populations are expected to differ in genetic composition. The frequencies of adaptive alleles vary in space or in different environments [51]. Accordingly, the expression levels of these genes vary and affect population adaptation. If the gene frequencies follow a clinal pattern, the optimum value of population gene expression level (*z*_opt_) is also expected to vary along a gradient under additive inheritance. The plastic component of gene expression could modify the clinal pattern. Suppose that the expression level (*z*) of an individual at a specific gene in the *i*-th site is decomposed in the linear form as Equation (8). These components at a given site can be estimated, and each component follows a normal distribution:$s_{i}\sim N({\bar{s}}_{i},\;\sigma_{s}^{2}$), $g_{j}\sim N({\bar{g}}_{j},\;\sigma_{g}^{2}$), ${\mathrm{sg}}_{\mathrm{ij}}\sim N({\bar{\mathrm{sg}}}_{\mathrm{ij}},\;\sigma_{\mathrm{gs}}^{2}$), and $e_{\mathrm{ijk}}\sim N(0,\;\sigma_{e}^{2}$) where the symbols with bar hats are the means and $\sigma_{.}^{2}$ is the variance. Suppose that the optimum expression level (*z*_opt_) in the population can be decomposed in the same way as Equation (8). Let $Z={\left(s_{i},g_{j},{\mathrm{sg}}_{\mathrm{ij}}\right)}^{\prime }$ be the vector and ***Z***_opt_ be the vector with corresponding optimum components. The fitness of an individual with the gene expression level *z* is expressed as

(9) $$w\left(Z\right)=\exp\left(-\frac{{\left(Z-Z_{\mathrm{opt}}\right)}^{\prime }\left(Z-Z_{\mathrm{opt}}\right)}{2V_{s}}\right)$$

where *V_s_* indicates the selection strength. This is the stabilizing selection towards an optimum expression level at the *i*-th site or environment for a single gene. Assume that an individual with ***Z*** follows a normal distribution, with density function *ϕ*(***Z***). Thus, the ${\bar{r}}_{g}$ of the population is generally expressed as

(10) $${\bar{r}}_{g}=\int w\left(Z\right)\phi \left(Z\right)dZ$$

where the genotype-by-site effects are included. Here, Equation (10) is not further explored but will be given elsewhere. A few studies have shown the presence of correlations between gene expression diversity and genetic diversity. In *Pseudotaxus chienii*, the gene expression diversity within and among populations was inversely related to nucleotide diversity [87]. Liu et al. [86] observed that the gene expression diversity was negatively correlated with population genetic distance or nucleotide diversity in *Cephalotaxus oliveri*, implying that the plastic responses were not significant.

**Table 2:** Associations between gene expression and environmental gradients in plants.

Species	Environmental Gradient	Study Design	Replication Level	Phenotype/Fitness Measured	Sample Tissue	Reference
*Primula sikkimensis*	Elevation and temperature	Transplant experiment across three elevational sites	3 biological replicates per site	Growth (sprouting, height) and reproductive traits (flower and seed number)	Leaves	[124]
*Artemisia californica*	Precipitation and drought	Field common-garden rainfall manipulation using two source populations (50% vs. 125% ambient precipitation)	3 biological replicates per population and rainfall treatment	Physiological and growth traits (photosynthesis, water use efficiency, leaf nitrogen, stem diameter, canopy volume)	Roots; leaves	[125]
*Suaeda salsa*	Salinity	Controlled hydroponic salinity treatment (0, 200, 500 mM NaCl)	3 biological replicates per treatment	No direct phenotype or fitness measurement	Roots; leaves	[126]
*Helianthus annuus*	Latitude	Common-greenhouse experiment using six source populations	4 biological replicates per population (20 samples after QC)	Seed oil composition (fatty acid saturation)	Seeds	[127]
*Forsythia suspensa*	Drought	Common-garden drought experiment (80% vs. 20% soil water content)	3 biological replicates per population and drought treatment	Physiological drought-tolerance traits (proline, soluble sugars, malondialdehyde)	Leaves	[128]
*Coffea mauritiana*	Climate	Eight populations grown in a long-term common garden	3 biological replicates per population (individual trees)	Leaf morphology and cuticular wax composition	Leaves	[129]
*Picea abies*	Latitude and light quality	Controlled-environment SUN/SHADE experiment using four latitudinal populations	3 biological replicates per treatment for the northernmost and southernmost populations	Germination and hypocotyl elongation under shade	Whole seedlings	[130]
*Quercus petraea*	Elevation and temperature	Natural-population sampling across two valleys and three elevations at two dormancy stages	2 biological replicates per population and dormancy stage	Bud phenology (leaf unfolding date)	Buds	[123]
*Rhododendron sanguineum* var. *haemaleum*	Elevation	Natural-population sampling along four elevations	3 biological replicates per site and organ	No direct phenotype or fitness measurement	Flower buds; leaf buds	[121]
*Larix sibirica*	Elevation	Natural-population sampling from 12 plots along the Altai Mountains, with all samples collected at 10 a.m.	3 biological replicates per plot	No direct phenotype or fitness measurement	Needles	[110]
*Rhododendron anthopogon*	Elevation	Natural-population sampling at three sites along an elevational gradient (3200, 3500, and 3900 m)	3 biological replicates per site	No direct phenotype or fitness measurement	Leaves	[131]

### 3.3. Regulation by Genomic Structural Variants

#### 3.3.1. Structural Variants and Transposable Elements

Genomic structural variants (SVs) are prevalent and play important roles in speciation and environmental adaptation [132,133]. SVs are usually defined as genomic segments larger than 50 bp in length, including duplications, inversions, translocations, and complex SVs [134]. Compared with SNPs, SVs could have larger effects on plant adaptation [135]. Their formation mechanisms include non-allelic homologous recombination (NAHR), non-homologous end joining (NHEJ), replication-based mechanisms, and the activity of transposable elements (TEs). SV types and sizes are often associated with diverse selective and evolutionary processes, including positive, negative, balancing selection, and neutral or nearly neutral processes [136]. A pangenome analysis of *Quercus variabilis* indicated that SV- and SNP-based scans revealed distinct selection signals, with only a few overlaps between the candidate regions [137]. Hämälä et al. [138] found that many SVs in *Theobroma cacao* were under selective constraints, which limits adaptation, whereas a small number of SVs affected adaptation to heterogeneous environments by altering gene expression. Regulatory effects of SVs were also reported in *Brassica napus* [139]. A pangenome analysis of *Arabidopsis thaliana* showed that more than 50% of large SVs (>500 bp) originated from TE insertions, and these TE-derived variants contributed to habitat adaptation [140]. Although few studies directly connected SVs to plant species’ range, the above cases imply that SVs can participate in regulating ${\bar{r}}_{g}$ in the edge populations.

TEs are involved in regulating plant gene expression and adaptation but also exhibit distinct regions covered by SNPs-associated adaptation. Wang et al. [141] showed that SINE transposons promoted the accumulation of beneficial metabolites in Himalayan *Prunus* species and enhanced adaptation to harsh habitats. Zhao et al. [142] identified a Helitron transposon in *Populus tomentosa* populations. This transposon reduced *WRKY* expression and contributed to adaptation. In addition, TEs and SNPs provide complementary sources of genetic variation underlying plant adaptation to environmental heterogeneity. Rogivue et al. [143] showed little overlap between SNP- and TE-associated markers of local adaptation in *Arabis alpina*, suggesting that these variants could reflect distinct candidate genes and biological functions in adaptive responses. Castanera et al. [144] showed that transposon insertion polymorphisms (TIPs) captured more expression-associated genomic signals than SNPs alone in *Oryza sativa* ssp. *indica* and *O. sativa* ssp. *japonica*. When both were associated with the same expression trait, TIPs typically accounted for a greater fraction of expression variation.

In addition, TEs may be involved in plant spatial distribution. For instance, TE insertions contributed to adaptation to environmental changes in *Phaseolus vulgaris* and *P. lunatus* with wider-range expansions [145,146]. In *Capsella rubella*, the frequent insertions of TEs into the *FLC* region affected *FLC* gene expression and explained approximately 12.5% of natural variation in flowering time [147]. During the expansion of *Zea mays* into high-latitude regions, two independent TEs upstream of *ZmCCT9* and *ZmCCT10* reduced flowering repression, increased photoperiod insensitivity, and facilitated adaptation to high-latitude environments [148]. Hämälä et al. [149] observed that more abundant TEs in populations of *Arabidopsis lyrata* at higher altitude increased their adaptation. These examples imply that TEs participate in shaping a species’ range.

#### 3.3.2. Gene Family and Genome Size

Most plants and animals have experienced partial- or whole-genome duplications in their long-term evolutionary history. Subsequent species divergences between close relatives often come from gene family expansion and contraction as well as genome size changes. The changes in gene family size could affect plant adaptation to different environments [150]. For instance, Yu et al. [151] identified 179 significantly expanded gene families in *Acer catalpifolium*, including stress-related families such as the FRS–FRF gene family, which enhanced adaptation to environmental stress. The rapid expansion of the *WRKY* gene family in *Primulina huaijiensis* was associated with adaptation to limestone karst environments [152]. Comparison of nine *Rhododendron* genomes showed that disease-resistance-related families were expanded in *R. neriiflorum*, a species from warm and humid subalpine forests, but contracted in *R. nivale* and *R. redowskianum*, which occur in cold alpine meadow and tundra environments with lower microbial diversity [153]. These examples imply that the changes in gene families potentially influence ${\bar{r}}_{g}$ and hence a species’ range.

Differences in genome size and geographic range size between close relatives could likely imply the role of genome size variation. Compared with the wider-range relatives, the narrower-range species usually have smaller *N_e_*, and lower genetic diversity due to genetic drift and inbreeding depression [154,155,156]. Further potential functional gene loss or gene family contraction weakens their ability to respond to environmental fluctuations (Table 3). The large genome constraint hypothesis predicts that angiosperms with larger genomes have lower dispersal abilities and hence narrower ecological niches and smaller geographic ranges [17]. Yet this prediction is not universal. Some narrow-range species have smaller genomes than their widespread relatives (Table 3), which may reflect genomic streamlining and functional specialization under specific selective pressures. The widespread *Acer davidii* has more expanded gene families and fewer contracted gene families than *A. tsinglingense*, suggesting that genomic redundancy may facilitate adaptation to diverse habitats. In contrast, the endangered and narrowly distributed *A. tsinglingense* may be adapted to small habitats [100].

Although whole-genome duplication (WGD) occurs less frequently than tandem duplication [165], WGD or polyploidization represents an important evolutionary process that can promote plant diversification, speciation, and adaptation to new environments [166,167]. For example, the tetraploid *Allium przewalskianum* extends farther west and south than the diploid populations in the Qinghai–Tibet Plateau, suggesting broader ecological adaptability in tetraploids [168]. A similar relationship between polyploidy and range expansion was recorded in *Solidago canadensis* [169]. Similarly, the polyploid plants show higher environmental tolerance than their diploid relatives, especially under drought and salt stress [170,171], as reported in *Pugionium* and *Brachypodium hybridum* [172,173]. However, polyploidy plants do not always confer an advantage in extreme environments, such as in *Chamaenerion angustifolium* [174]. Taken together, these cases update reports showing that changes in genome size influenced plant species ranges.

Notice that the preceding discussions provide preliminary conclusions regarding the roles of SVs, TEs, gene families and genome sizes in shaping a species’ range. Several other factors, such as assembly methods and annotation pipelines, could affect these comparisons. Thus, more rigorous comparisons and meta-analyses are needed before a general conclusion is drawn across many plant species.

## 4. Detecting Adaptive Signatures by *K_a_*/*K_s_*

**Figure 2:**
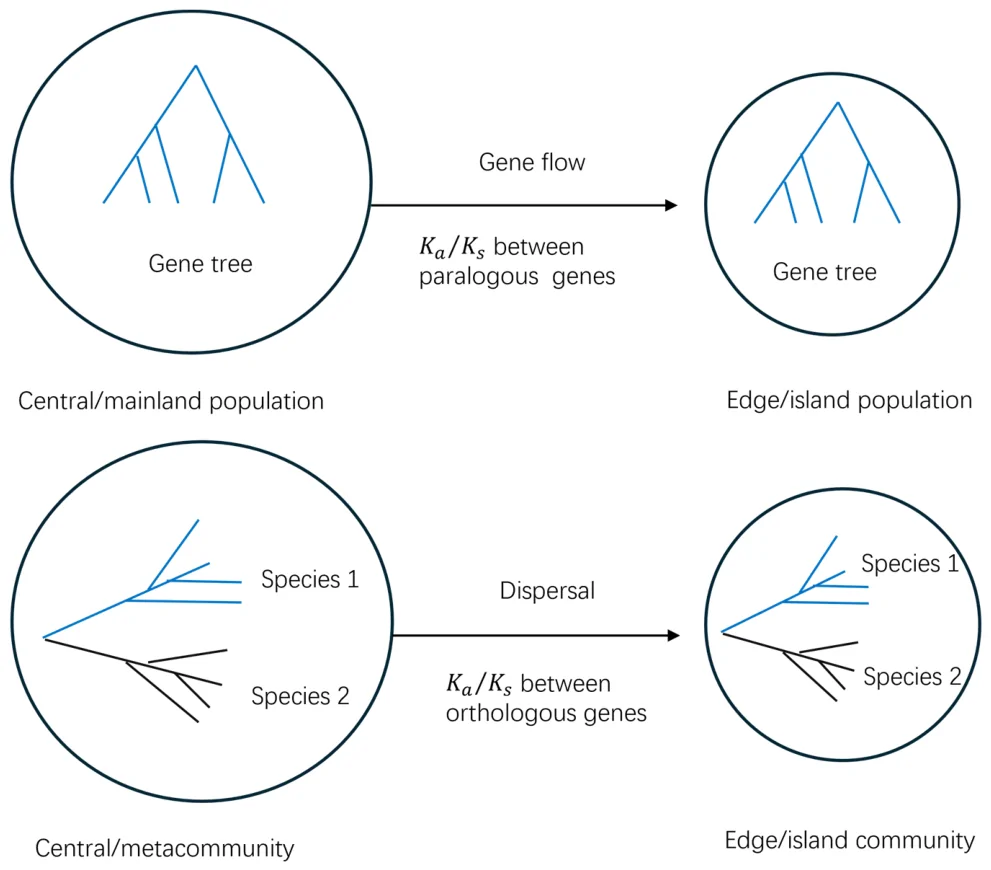
Detecting adaptive signatures at the species’ edge from the comparison of *K_a_*/*K_s_* between the central and edge populations (**upper** panel for intraspecific competition) or between the central and edge communities (**lower** panel for interspecific competition). Note that the interacting species in a community are previously identified before analysis of adaptation detection.

A species’ range may be governed by joint effects of selection, gene flow, mutation, and genetic drift. The edge populations often maintain smaller *N_e_* where genetic drift is intensified, and selection efficacy is reduced. As a result, deleterious mutations accumulate more readily, which slows or constrains range expansion [42]. For instance, the European subspecies *Arabidopsis lyrata* ssp. *petraea* expanded northward after the Last Glacial Maximum, yet its edge populations experienced a sharp reduction [111]. The adaptive differences between the edge and central populations could be inferred from comparing their differences in molecular evolutionary rates (Figure 2). Strong purifying selection in the edge population indicates a reduced potential for further range expansion.

Kryazhimskiy et al. [175] derived the ratio between the nonsynonymous substitution rate (*K_a_*) and the synonymous substitution rate (*K_s_*) for an adaptive gene in a single population:

(11) $$\frac{K_{a}}{K_{s}}=\frac{D(2\mathrm{Ns},4N_{e}\mu )}{D(0,4N_{e}\mu )}$$

The function *D*(2*Ns*, 4*N_e_μ*), *D*(0,4*N_e_μ*) and their calculations were described in Kryazhimskiy et al. [175]. Notice that *D*(2*Ns*, 4*N_e_μ*) and *D*(0,4*N_e_μ*) measure the polymorphisms due to the non-silent mutations per non-silent site and the silent mutations per silent site, respectively. The ratio within populations refers to the polymorphic sites where mutant alleles are not fixed in the short term. Kryazhimskiy et al. [175] showed that, in a single population, purifying selection is indicated with *K_a_*/*K_s_* < 1, whereas positive selection is indicated with *K_a_*/*K_s_* > 1. The ratio *K_a_*/*K_s_* increases as positive selection becomes stronger, but the increase becomes progressively smaller under strong positive selection, which differs from the pattern of *K_a_*/*K_s_* under interspecific competition. Stronger purifying selection implies the presence of highly deleterious alleles, which impedes adaptation of the edge populations. Gene flow from the central populations reinforces restriction of a species’ range [9,50].

Empirical studies were recorded for the intraspecific *K_a_*/*K_s_* analysis. Wang & Heckel [176] showed that *Microtus arvalis* subsp. *orcadensis* had a higher *K_a_*/*K_s_* than its close continental relative *Microtus arvalis*. The elevated *K_a_*/*K_s_* was mainly attributed to the smaller *N_e_* maintained in the edge populations. However, higher molecular evolutionary rates were also reported for the continental populations than for the island populations [177], which arose from more efficient positive selection in the large populations [178]. In addition, molecular evolutionary rates were also correlated with the environmental gradients, such as a positive correlation with temperature or a negative correlation with latitude in ectotherms [179], a negative correlation with altitude in *Sus scrofa* [180], but a positive correlation with altitude in *Rhododendron* [181].

Adaptive signatures at the edge of a species could potentially be detected at the community level where interspecific interactions (competition and coexistence) differ between the edge and central communities. This is essentially consistent with Darwin’s viewpoint where a species’ range is determined by both abiotic and interspecific competition [8]. Suppose that species associations have been previously identified using community ecological methods. There are several methods that can be used to detect species associations in a community [182,183]. A phylogenetic tree can be constructed in the central community. Similarly, a phylogenetic tree in the edge community in which the edge population of a focal species is embedded can be constructed as well (Figure 2). The *K_a_*/*K_s_* ratio can be estimated from orthologous genes shared by interacting species [106]. Assume that the *A* allele has a constant selective advantage *s* over the *a* allele at a biallelic locus in a randomly mating population of effective size *N_e_*. According to Kimura [184], *K_a_*/*K_s_* is given by

(12) $$\frac{K_{a}}{K_{s}}=\frac{4N_{e}s}{1-e^{-4N_{e}s}}(\mathrm{positive}\text{-}\mathrm{selection genes},s>0)$$

(13) $$=\frac{4N_{e}s}{e^{4N_{e}s}-1}(\mathrm{negative}\text{-}\mathrm{selection genes},s<0),$$

where *s* is the selection coefficient of mutant alleles. The *K_a_*/*K_s_* ratio refers to different alleles that are fixed in alternative lineages after a long-term history.

It is hypothesized that the species interaction in the edge community could cause the *K_a_*/*K_s_* ratio to be biased from its value in the central community. When the associated species imposes constraints on the focal species, this probably reduces its *r_e_*(*N*_1_, *N*_2_) and its effective population size (*N_e_*), which could increase the *K_a_*/*K_s_* ratio of deleterious genes. Similarly, positive interspecific interactions could increase *r_e_*(*N*_1_, *N*_2_) and the effective population size of the focal species, which could enhance the *K_a_*/*K_s_* ratio. Thus, a comparison of *K_a_*/*K_s_* between the central and edge communities could likely reflect adaptation signatures in the edge populations. This hypothesis needs to be verified with data collection.

Kapralov [185] estimated the *K_a_*/*K_s_* ratio of 27 species of the genus Schiedea (Caryophyllaceae) sampled from the Hawaiian Islands and the mainland. The higher ratio in the island group than in the mainland group could reflect relaxed purifying selection in the smaller island community. More positive selection in the mainland group reflects interspecific competition in the mainland community. Community size influences species interactions *r_e_*(*N*_1_, *N*_2_, …), which could in turn influence the effective population size of a focal species and then genetic drift effects and the *K_a_*/*K_s_* ratio [186].

Other empirical studies have compared interspecific *K_a_*/*K_s_* across their spatial distributions. For example, in four high-elevation frog species from the Qinghai–Tibet Plateau, genes with high *K_a_*/*K_s_* were annotated to participate in key pathways related to energy metabolism, mitochondrial function, and DNA repair [187]. In two alpine *Lobelia* species, Zhao et al. [188] detected that genes associated with cold response, DNA repair, and gene expression regulation were under positive selection. Liu et al. [181] detected genes under positive selection in *Rhododendron* distributed in elevation regions. Whether the pattern of *K_a_*/*K_s_* ratio is associated with interspecific interaction remains to be determined.

## 5. Regulation by Mating Systems

Alternation of generations and sexual reproduction are important innovations in eukaryote evolution. Selfing and outcrossing represent two different strategies of gene transmission across generations. Selfing can provide reproductive assurance and partly retains the functions that are equivalent to those of clonal or asexual reproduction. Outcrossing, by contrast, promotes genetic mixing among individuals. The mixed mating system combines both strategies and allows populations to balance the reproductive assurance and the maintenance of genetic variation. The life-cycle pattern differs widely among plant lineages, with the relative lengths of the gametophyte vs. sporophyte phases generally decreasing in the order of algae, bryophytes, ferns, gymnosperms, and angiosperms. Accordingly, the genetic loads could come from each phase and are unequal between the two phases, which shapes plant adaptation and species’ ranges. Previous studies have addressed how mating systems affect genetic diversity and a species’ range, as well as theoretical relationships between mating systems and molecular evolution [51,189]. In Section 2, we discussed how mixed mating systems regulate ${\bar{r}}_{g}$. Here, we expand discussions on the role of selfing in regulating molecular evolution and adaptation in the edge populations.

Selfing facilitates the maintenance of deleterious mutant alleles and reduces population fitness. However, this conclusion depends on certain conditions. Xiao et al. [53] proposed the concept of a critical selfing rate α^*^ for large populations. A fraction of deleterious alleles can be fixed and generate genetic load when the selfing rate exceeds α^*^ under mutation-selection processes. Assume that the fitness in the gametophyte phase is 1 − *s*_h_ for the ancestral allele (*A*) and 1 for the mutant allele (*a*) in both pollen and ovules. The fitness in the sporophyte phase is 1 for the *AA* genotype, 1 − *hs*_d_ for *Aa*, and 1 − *s*_d_ for *aa*. In small populations with genetic drift effects, *K_a_*/*K_s_* is shaped by the selfing rate *α*. According to Equation (14) of Li et al. [189], when only gametic selection occurs, *K_a_*/*K_s_* is given by

(14) $$\frac{K_{a}}{K_{s}}=\frac{-4N_{e}\left(1-\frac{\alpha }{2\cdot \alpha }\right){\cdot 2s}_{h}}{1-\exp\left(-4N_{e}\left(1-\frac{\alpha }{2-\alpha }\right)\cdot 2s_{h}\right)}$$

When *s*_h_ > 0, the mutant allele is beneficial. When *s*_h_ < 0, the mutant allele is deleterious. When only zygotic selection occurs under a linear additive selection model (*h* = 1/2), according to Equation (17) of Li et al. [189], *K_a_*/*K_s_* is expressed as

(15) $$\frac{K_{a}}{K_{s}}=\frac{4N_{e}\frac{1}{2-\alpha }s_{d}}{\exp\left(4N_{e}\frac{1}{2-\alpha }\right)-1}$$

The mutant allele is beneficial when *s*_d_ < 0, but deleterious when *s*_d_ > 0.

**Figure 3:**
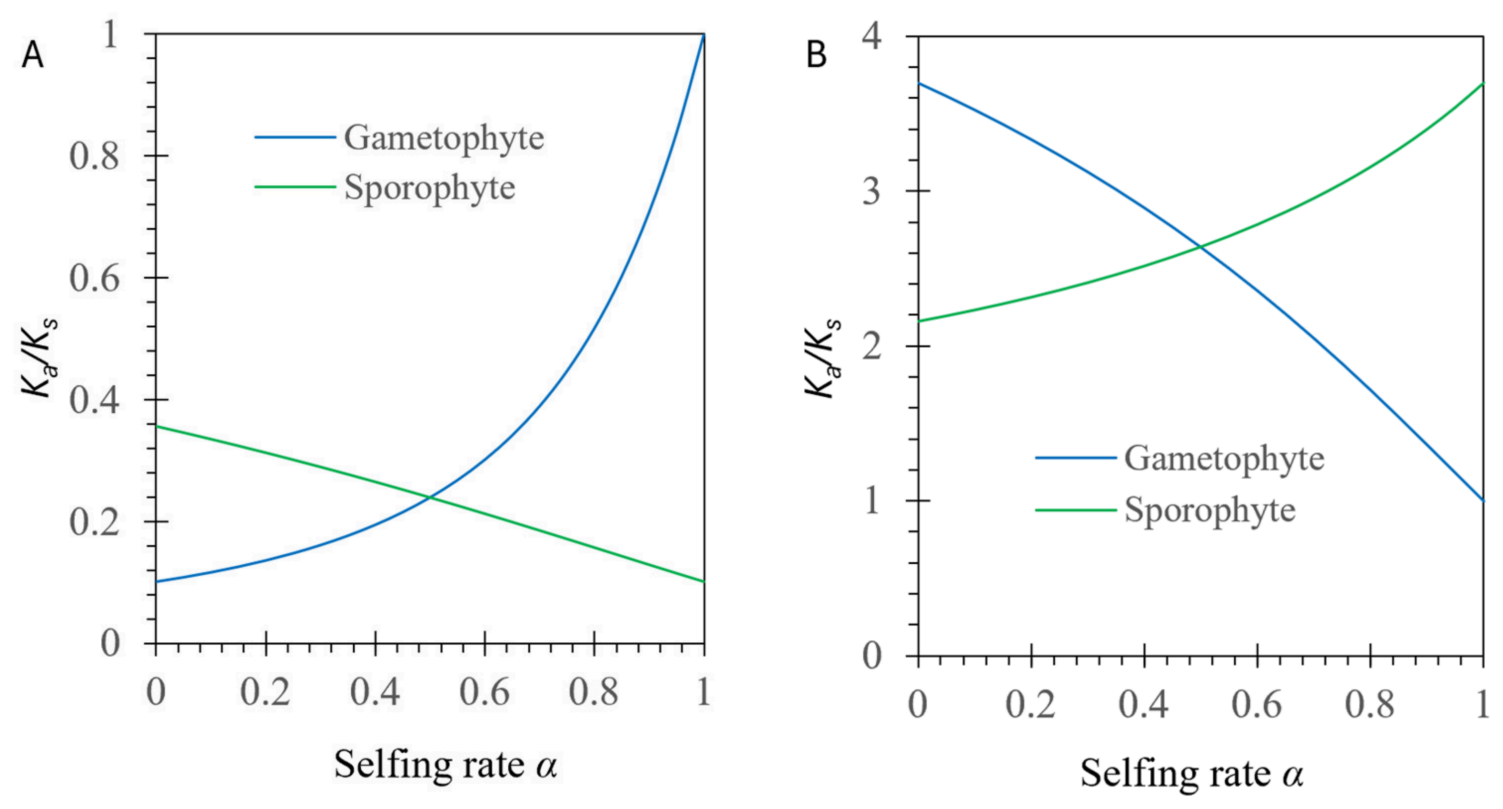
Examples of selfing regulating the molecular evolutionary rates (*K_a_*/*K_s_*). (**A**) Deleterious mutant genes expressed in the gametophyte phase only (blue line) and in the sporophyte phase only (green line), parameterized with *s*_h_ = −0.015, *s*_d_ = −0.03, and *N*_e_ = 30; and (**B**) Beneficial mutant genes expressed in the gametophyte phase only (blue line) and in the sporophyte phase only (green line), parameterized with *s*_h_ = 0.015, *s*_d_ = 0.03, and *N*_e_ = 30. The results are derived from Equations (14) and (15).

When the mutant allele is deleterious (Figure 3A), selfing reduces the efficiency of gametic selection. Genes expressed only at the gametic stage are therefore subjected to relaxed selection, and *K_a_*/*K_s_* tends to unity. By contrast, genes expressed only at the zygotic stage are subjected to stronger purifying selection, leading to a lower *K_a_*/*K_s_*. A contrast pattern is predicted for beneficial mutant alleles (Figure 3B). Selfing weakens gametic selection, so genes expressed in the gametophyte phase only have *K_a_*/*K_s_* closer to one. However, genes expressed in the sporophyte phase are subject to stronger positive selection, leading to a higher *K_a_*/*K_s_*. Selfing regulates molecular evolution rates in opposite directions depending on whether genes are expressed in the gametic or zygotic phase. This phase-specific effect provides a theoretical explanation for how mating systems shape species adaptation to environments and hence the species’ ranges.

Species of self-fertilization often have smaller genomes but larger geographic ranges [17]. The edge populations may receive genetic inputs from the central or other populations. Deleterious mutations therefore accumulate during spatial expansion. Some species exhibit a partial selfing system in the edge populations, such as *Arabidopsis lyrata* ssp. *lyrata* at the edge [43]. This transition facilitates the generation of unequal genetic loads between the gametic and zygotic phases.

Assume that the fitness in the gametophyte phase is 1 for the ancestral allele (*A*) and 1 − *s*_p_ for the mutant allele (*a*) in pollen and 1 − *s*_o_ in ovules. The fitness in the sporophyte phase is 1 for the *AA* genotype, 1 + *s*_het_ for *Aa*, and 1 + *s*_hom_ for *aa*. Xiao et al. [53] provided the density function of mutant-allele frequency (Equation (10)) and the mean fitness at both phases under a mixed mating system. Based on these results, and assuming a linear additive selection model (*s*_het_ = *s*_hom_/2), the frequency density of the deleterious mutant gene expressed in the gametophyte phase only is given by

(16) $$\phi \left(q\right)=\frac{4N_{e}\mu }{q(1-q)}\frac{1-\exp\left(2N_{e}\left(1-\frac{\alpha }{2-\alpha }\right)s_{\mathrm{hap}}(1-q)\right)}{1-\exp\left(2N_{e}\left(1-\frac{\alpha }{2-\alpha }\right)s_{\mathrm{hap}}\right)}$$

where *μ* is the mutation rate from the ancestral allele to the deleterious allele and *s*_hap_ is equal to *s*_p_ + *s*_o_. The frequency density of the deleterious mutant gene expressed in the sporophyte phase only is given by

(17) $$\phi \left(q\right)=\frac{4N_{e}\mu }{q(1-q)}\frac{1-\exp\left(-4N_{e}\frac{1}{2-\alpha }s_{\mathrm{hom}}(1-q)\right)}{1-\exp\left(-4N_{e}\frac{1}{2-\alpha }s_{\mathrm{hom}}\right)}.$$

The ratio of genetic load in the gametic phase ($L_{\mathrm{hap}}=\int_{0}^{1}s_{\mathrm{hap}}\mathrm{q\phi}\left(q\right)\mathrm{dq}$) to that in the zygotic phase ($L_{\mathrm{dip}}=-\int_{0}^{1}s_{\mathrm{hom}}\mathrm{q\phi}\left(q\right)\mathrm{dq}$) is derived below:

(18) $$\frac{L_{\mathrm{hap}}}{L_{\mathrm{dip}}}=-\frac{s_{\mathrm{hap}}}{s_{\mathrm{hom}}}\cdot \frac{1-\exp(b)}{1-\exp(a)}\cdot \frac{\int_{0}^{1}{\left(1-q\right)}^{-1}(1-\exp\left(a\left(1-q\right)\right)\mathrm{dq}}{\int_{0}^{1}{\left(1-q\right)}^{-1}(1-\exp\left(b\left(1-q\right)\right)\mathrm{dq}}$$

where $a=\left(1-\frac{\alpha }{2-\alpha }\right)\cdot 2N_{e}s_{\mathrm{hap}}$ and $b=-\frac{1}{2-\alpha }\cdot 4N_{e}s_{\mathrm{hom}}$. Notice that the mean Malthusian population fitness ${\bar{r}}_{g}$ is equivalent to $\log(\bar{w})$ where $\bar{w}$ is the mean population fitness in Wright’s definition. Consider genes expressed in a single phase only (gametophyte or sporophyte phase). The genetic component ${\bar{r}}_{g}$ in the Malthusian fitness is ${\bar{r}}_{g}=\ln\left(1-L_{\mathrm{hap}}\right)+\ln\left(1-L_{\mathrm{dip}}\right)\approx -L_{\mathrm{hap}}-L_{\mathrm{dip}}$.

**Figure 4:**
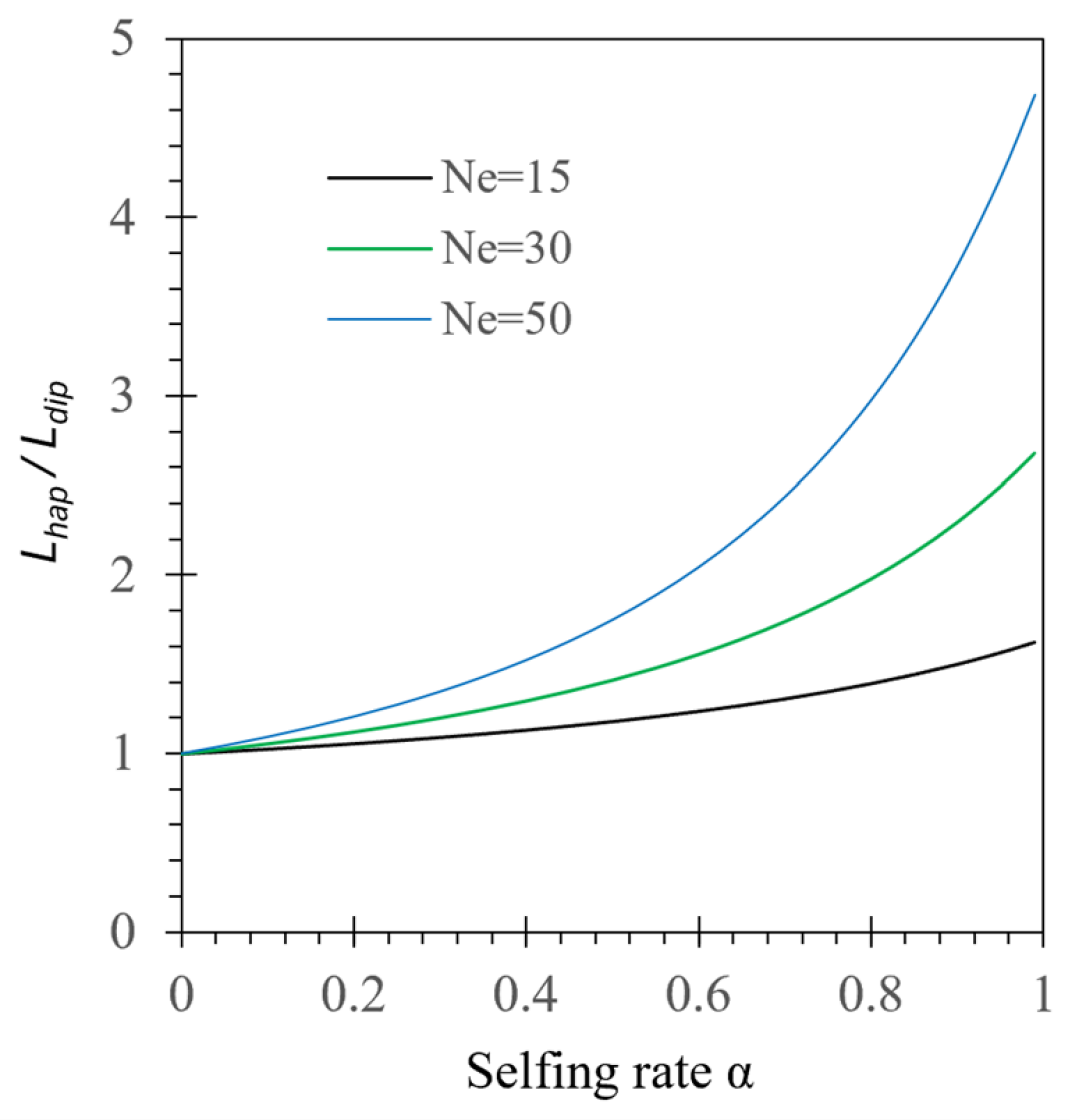
Examples of the relative genetic loads between the gametic and zygotic phases (*L*_hap_/*L*_dip_) regulated by the mating system, commonly parameterized with *s*_hap_ = 0.03 and *s*_hom_ = −0.03. The genetic drift effects were compared under three effective population sizes: *N_e_* = 15 (black lines), 30 (green line) and 50 (blue line). The results were derived from Equation (18), where the integral calculation was obtained using the NIntegrate function of Mathematica v 9.0 [190], with the frequency (*q*) in the range [10^−6^, 1].

Assume that the mutant alleles are deleterious in the gametophyte phase (*s*_hap_ > 0) or in the sporophyte phase (*s*_hom_ < 0) according to the fitness settings. Under the same selection strength, the mean genetic load in the gametic phase (*L*_hap_) gradually exceeds that generated in the zygotic stage (*L*_dip_) as the selfing rate increases (Figure 4). The genetic drift effects do not alter the general pattern since a positive partial differential of *L*_hap_/*L*_dip_ with respect to the selfing rate holds, i.e., $\partial \left(\frac{L_{\mathrm{hap}}}{L_{\mathrm{dip}}}\right)/\partial \alpha >0$. It is hypothesized that the edge populations could possess different mating systems to regulate the total genetic load (*L*_hap_ + *L*_dip_) so as to adapt to habitats. This hypothesis remains to be tested by empirical studies.

Ample evidence addresses the relationships between the mating system and population adaptation at the edge. The type of mating system can alter species’ ranges [33,191]. A transition from outcrossing to selfing reduces the efficiency of natural selection and allows mildly deleterious mutations to accumulate, which limits population adaptation to new environments. This could increase *K_a_*/*K_s_* and the genetic load at the gametic phase, which is rarely emphasized in the literature. Such patterns also likely occur in organelle genomes [192]. Both theoretical and empirical studies indicate that self-fertilization can mitigate genetic load at the edge through range expansion [193,194]. However, selfing does not fully prevent the accumulation of expansion load [195]. It should be noted that mating systems do not necessarily change during range expansion. Many species retain their ancestral mating systems despite the presence of the expansion load [111,112,196].

## 6. Conclusions and Perspectives

Selection and dispersal are two dominant processes affecting a plant species’ range, although other evolutionary processes cannot be excluded. Current availability of population genome data provides new sources to explore evolutionary mechanisms underlying a species’ range at a more fundamental level. Here, we explored the eco-evolutionary model to interpret species’ range based on the general diffusion–reaction framework and reviewed recent advances in molecular studies from the perspectives of population genomics, transcriptomics, and genomic structural variation. Specific conclusions are summarized below:

(1) The mean Malthusian fitness $\bar{r}$ in the edge populations can be uniformly decomposed into the genetic component ${\bar{r}}_{g}$ (hard selection), ecological component *r_e_* (soft selection) and their potential genetic-by-environment interactions (${\bar{r}}_{\mathrm{ge}}$). The ${\bar{r}}_{g}$ may come from different genetic loads generated by four basic evolutionary forces, while the *r_e_* may come from temporal environmental variation, density dependence, and interspecific interactions. The relative strengths of these two types of regulation remain to be quantified across plant species.
(2) Landscape genomic analysis is currently applied to identify genome-wide SNPs associated with adaptation. This method combines multiple levels of data (population genomic, environmental and geographical data) and identifies adaptation signatures at the genome-scale. It may help to reveal possible genetic processes regulating population growth (${\bar{r}}_{g}$) but has not been well linked to the formation of a species’ range.
(3) RNA-seq data and expression plasticity $\left({\bar{r}}_{\mathrm{ge}}\right)$ are rarely applied to interpret a species’ range. The nature of this type of data is its sensitivity to changes in abiotic and biotic environmental factors and promises the hope of predicting species adaptation. Gene expression data helps to reveal the genetic component of population growth rate $\left({\bar{r}}_{g}\;+\;{\bar{r}}_{\mathrm{ge}}\right)$.
(4) Although some genomic structural variants (SVs) and transposable elements (TEs) are associated with population adaptation, their connections to a species’ range are rarely studied. Genome size and gene family expansion/contraction also affect species ranges, although most support comes from comparisons of species with different range sizes. Whether these impacts are present across multiple plant species awaits more data collection.
(5) The idea of using estimates of *K_a_*/*K_s_* to detect adaptive signatures in the edge populations needs data support. Divergent selection could be inferred through comparisons between the central and edge populations or between the central and edge communities in terms of the *K_a_*/*K_s_* ratio. This line of study remains to be explored.
(6) The mating systems provide a way to regulate genetic loads in the edge populations. Selfing reduces the efficiency of gametic selection, promotes the accumulation of deleterious mutations, and increases the proportion of the total genetic load from the gametic stage. Selfing also strengthens selection at the zygotic stage and reduces the genetic load from this stage. Small edge populations could weaken this regulatory effect. Plants with different lengths of gametophyte phase in a life cycle could evolve different ways to regulate a species’ range. This awaits empirical evidence.

Apart from the preceding conclusions, the following questions deserve emphasis in future studies.

(1) The eco-evolutionary model could be extended by including gene expression traits as quantitative traits in modeling selection and adaptation. This provides a basis for directly incorporating molecular traits into theoretical models of species distribution. Such work may provide insights into the molecular mechanisms that regulate adaptation in the edge populations.
(2) Population genomic analysis can detect diverse forms of genetic variation across the genome. A major concern lies in the methods that limit the accurate identification of all classes of structural variation. This methodological gap has constrained the application of SVs and TEs to ecological and evolutionary studies [133,197]. Although genome scans and environmental association analyses have identified candidate genes and genomic regions, most findings are confined to statistical associations. Direct evidence for the functional roles of candidate genes in adaptation is still scarce [198,199]. Further work is needed to clarify how genomic variation can be applied to explain species distribution patterns.
(3) A key challenge is to evaluate the relative strengths of ecological regulation or soft selection versus genetic regulation or hard selection. Molecular explanations of a species’ range mainly involve hard selection and emphasize genetic load and evolutionary processes. Ecological regulation operates over shorter timescales. The extent to which the two regulatory mechanisms shape a species’ range varies among species. Different modes of selection could generate contrasting outcomes, and range changes under hard selection may even lead to extinction [42]. More accurate predictions of a species’ range may depend on models that account for both hard and soft selection.

In general, future studies need to integrate multiple layers of data with complementary research methods. Multi-omics studies that combine population genomics with transcriptomics, epigenomics, metabolomics, and related data can reveal how genetic variation influences adaptation to environments. Experimental studies, including common-garden experiments, gene editing, and functional validation, would be effective in testing the adaptive roles of candidate genes and genomic structural variants. Linking molecular data with theoretical models would provide a deeper understanding of the molecular mechanisms underlying a plant species’ range.

## Figures and Tables

**Table 3:** Comparison between narrow- and wide-range relative species in genome and gene family size.

Narrow-Range Species	Genome Size (MB)	Gene Family Expansion (+)/Contraction (−)	Widespread Relative Species	Genome Size (MB)	Gene Family Expansion (+)/Contraction (−)	Reference
*Acer tsinglingense*	791.40	+1388/−2573	*Acer davidii*	1291.99	+4456/−709	[100]
*Platycarya longipes*	695.79	+45/−73	*Platycarya strobilacea*	703.509	+87/−23	[157]
*Corylus chinensis*	366.28	+371/−998	*Corylus heterophylla*	370.75	+672/−1017	[158]
*Prunus hongpingensis*	213.47	+218/−377	*Prunus armeniaca*	220	+984/−806	[159]
*Prunus zhengheensis*	209.82	+218/−380	*Prunus armeniaca*	220	+984/−806	[159]
*Dipteronia dyeriana*	914.77	+470/−426	*Dipteronia sinensis*	711.47	+761/−339	[160]
*Ostrya rehderiana*	366.3	+526/−1003	*Ostrya chinensis*	371.64	+880/−750	[161]
*Toona ciliata*	520	+470/−1	*Toona sinensis*	596	+31/325	[162]
*Descurainia millefolia*	216	+1087/−1469	*Descurainia sophioides*	195.6	+1253/529	[163]
*Neolamarckia macrophylla*	598.43	+208/−4	*Neolamarckia cadamba*	744.5	+42/−89	[164]

## Data Availability

No new data were created or analyzed in this study. Data sharing is not applicable to this article.
